# The role of ferroptosis in osteoporosis: a cellular perspective on osteoblast, osteoclast and osteocyte dysfunction

**DOI:** 10.3389/fendo.2026.1742656

**Published:** 2026-02-19

**Authors:** Aysha Bakari Murusuri, Jun Tang

**Affiliations:** Department of Endocrinology, Zhongnan Hospital of Wuhan University, Wuhan, China

**Keywords:** ageing, antioxidant, ferroptosis, osteogenic cells, osteoporosis, postmenopausal

## Abstract

Osteoporosis is characterized by an imbalance between bone resorption and bone formation, leading to the loss of both trabecular and cortical bone mass, ultimately resulting in an increased risk of fractures. Osteoporosis represents a major global health burden, predominantly affecting elderly individuals and postmenopausal women. With the continued growth of the aging population, the prevalence of osteoporosis is expected to increase, highlighting the urgent need for more effective therapeutic strategies. Ferroptosis, a recently characterized form of iron-dependent, non-apoptotic cell death, has emerged as an important mechanism contributing to the pathogenesis of osteoporosis. A better understanding of ferroptosis may therefore provide new insights into therapeutic development. This review summarizes current evidence regarding the role of ferroptosis in osteoporosis, with particular focus on its effects on osteoblasts, osteoclasts, and osteocytes, as well as the impact of aging, estrogen deficiency, diabetes, glucocorticoid exposure, and obesity on ferroptosis in osteogenic cells.

## Introduction

### Ferroptosis

**Figure 1:**
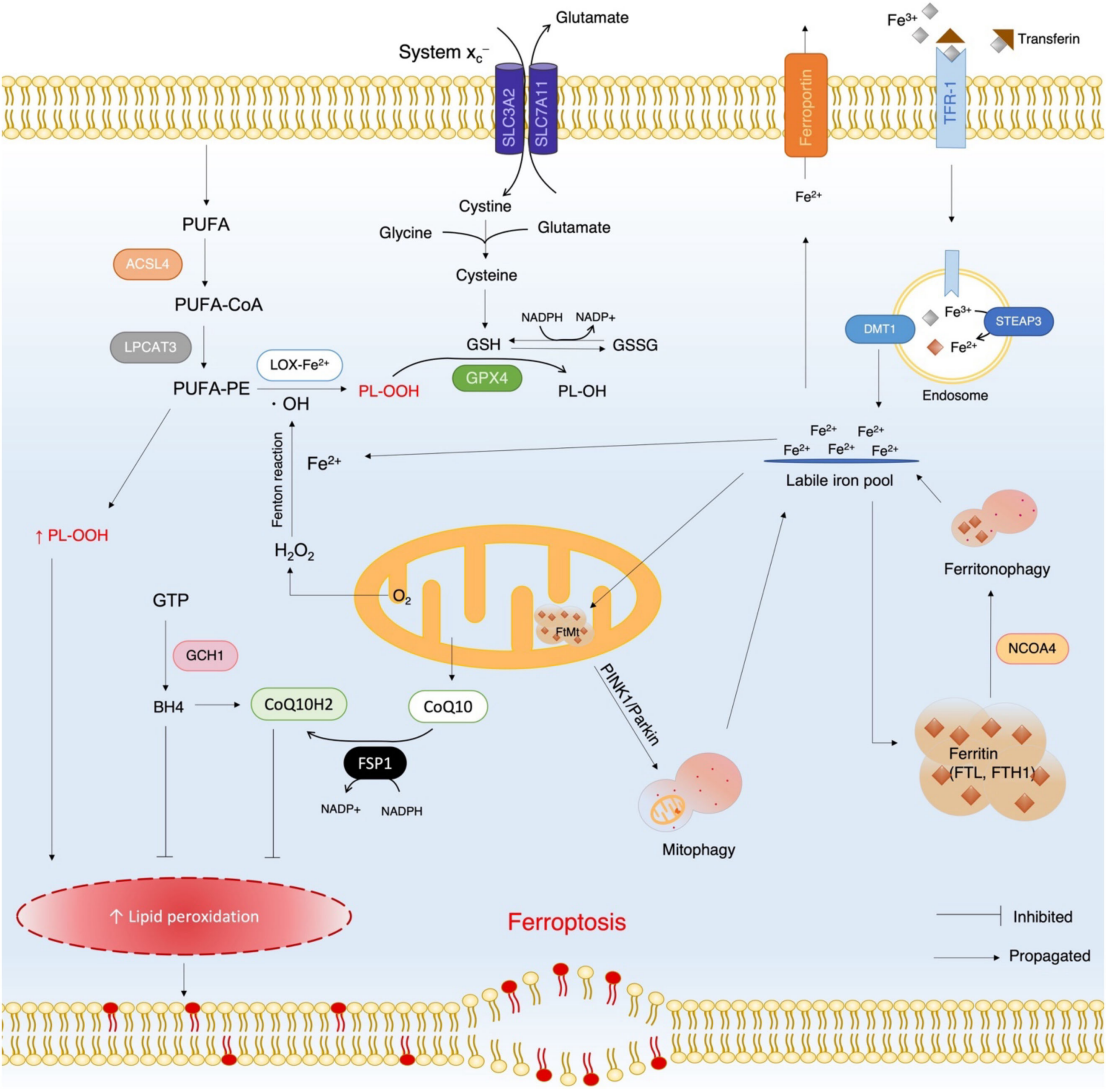
Schematic overview of the ferroptosis mechanism in cells. The right side of the diagram depicts iron metabolism including iron import, export, storage as ferritin, and its release via ferritonophagy. The left side illustrates the conversion of PUFAs into lipid peroxides, and the role of system xc- and GPX4 in reducing these peroxides into safer metabolites. The bottom left area of the diagram shows how accumulated lipid peroxides destabilize the cell membrane, alongside other antioxidant mechanisms that inhibit ferroptosis. TFR-1, transferrin receptor 1; STEAP3, six-transmembrane epithelial antigen of prostate 3 (a metalloreductase); DMT1, divalent metal iron transporter 1; NCOA4, nuclear receptor coactivator 4; FTL, ferritin light chain; FTH1, ferritin heavy chain 1; GPX4, glutathione peroxidase 4; GSH, reduced glutathione; GSSG, oxidized glutathione; LOX-Fe^2+^, lipoxygenase with iron cofactor; * OH, hydroxyl radical; PUFAs, polyunsaturated fatty acids; PE-PUFAs, phosphatidylethanolamine (PE)-bound polyunsaturated fatty acids; ACSL4, acyl-CoA synthetase long-chain family member 4; LPCAT3, lysophosphatidylcholine acyltransferase; PL-OOH, lipid peroxide; PL-OH, lipid alcohol; GTP, guanosine triphosphate; GCH1, GTP cyclohydrolase 1; BH4, tetrahydrobiopterin; CoQ10, ubiquinone 10; FSP1, ferroptosis suppressor protein 1; CoQ10H2, dihydroubiquinone 10.

The term ferroptosis was formally established in 2012 due to the recommendations of The Nomenclature Committee of Cell Death that recommended the classification of cell death to be based on molecular events rather than morphology (1, 2).Ferroptosis is a regulated, iron-dependent, non-apoptotic form of cell death that is driven primarily by phospholipid peroxidation within cellular membranes (3, 4). The central role of iron arises from its participation in the Fenton reaction, which generates highly reactive hydroxyl radicals, and from its function as an essential cofactor for lipid-peroxidizing enzymes such as lipoxygenases. Through these direct (radical-generating) and indirect (enzyme-cofactor) effects, iron promotes the accumulation of lipid peroxides that destabilize membranes and eventually cause cell rupture (**Figure 1**) (5). Molecules derived from lipid peroxides such as malondialdehyde (MDA), cause further cellular damage by reacting with proteins and nucleic acids (6, 7). Lipid peroxides originate largely from phosphatidylethanolamine (PE)-bound polyunsaturated fatty acids (PUFAs), which are generated by the sequential actions of acyl-CoA synthetase long-chain family member 4 (ACSL4) and lysophosphatidylcholine acyltransferase 3 (LPCAT3). PE-PUFAs are then oxidized either non-enzymatically by hydroxyl radicals or enzymatically by iron-dependent lipoxygenases (3, 7). Thus, hydroxyl activity as a direct effect of iron, while cofactor activity is an indirect effect of iron. Although iron can produce reactive oxygen species (ROS) and result in peroxidation, it is a necessary metal for various cellular functions. As a result, cells employ various antioxidant mechanisms, such as a glutathione-dependent pathway (3), and iron storage methods using cytosolic ferritin and mitochondrial ferritin (FtMt) to prevent oxidative stress (7, 8).

Glutathione is formed from glycine, glutamate, and cysteine (in the form of y-glutamyl cysteine). Cysteine is a derivative of cystine which is brought into the cell via a transmembrane protein antiporter named system x_c_^−^ (7). This antiporter is made up of two subunits, namely, solute carrier family 3 member 2 (SLC3A2) and solute carrier family 7 member 11 (SLC7A11), which are involved in the uptake of cystine and the simultaneous expulsion of glutamate. Glutathione is used in its reduced state (GSH) by the glutathione peroxidase 4 (GPX4) enzyme to convert lipid peroxide into lipid alcohol, which is a safer metabolite (3). Once used, GSH becomes oxidized glutathione (GSSG), which can be reduced back into GSH by the glutathione reductase enzyme in the presence of NADPH (7). Ferroptosis is triggered when this antioxidant defense becomes insufficient, typically due to excessive iron accumulation or impaired GSH synthesis, resulting in uncontrolled lipid peroxidation and catastrophic membrane failure (3, 5).

In addition to this canonical pathway, alternative antioxidant systems also modulate ferroptosis, including the ubiquinone-10 (CoQ10) pathway and the tetrahydrobiopterin (BH4) pathway (5). Ferroptosis suppressor protein-1 (FSP1) reduces CoQ10 to dihydro-CoQ10 (CoQ10H2), a potent lipid radical–trapping antioxidant, whereas BH4 is synthesized by GTP cyclohydrolase-1 (GCH1) and inhibits ferroptosis directly or indirectly by supporting CoQ10 biosynthesis (5, 9, 10).

Ferroptosis can be initiated through two major pathways—the extrinsic pathway and the intrinsic pathway. The extrinsic pathway is driven by extracellular or membrane-associated events, including increased iron uptake via transferrin receptor 1 (TFR1) or inhibition of the xc-cystine/glutamate antiporter system, leading to cysteine depletion and impaired glutathione synthesis. In contrast, the intrinsic pathway arises from intracellular disturbances, particularly the suppression or inactivation of antioxidant systems such as GPX4, resulting in unchecked lipid peroxidation. These pathways are modulated by diverse factors, including disease states, pharmacological inducers, cell regulatory programs, and iron chelators, ultimately converging on iron-dependent lipid peroxide accumulation and membrane damage (11).

There are several ways that ferroptosis can be initiated, with one being the introduction of inducers. Inducers, such as erastin, induce ferroptosis by inhibiting cysteine uptake by system x_c_^−^ (5). The p53 cell cycle regulatory factor p53 has the same effect by downregulating SLC7A11 transcription (12), which prevents GSH synthesis, resulting in lipid peroxides accumulation. Transcription factors, such as nuclear factor erythroid 2-related factor 2 (Nrf2), are crucial in regulating ferroptosis (13). In osteoblasts, ferroptosis is prevented by the Nrf2/heme oxygenase 1 (HO-1) pathway via activating HO-1, which upregulates GPX4 through regulation of antioxidant-responsive elements (ARE)- dependent genes (14, 15). Iron accumulation can be caused by increased uptake (exogenous source), decreased expulsion, or decreased storage (endogenous source). Some diseases, such as hemochromatosis cause increased iron accumulation due to decreased regulation by hepcidin. Iron is then taken up excessively by the cell via TFR1. Inversely, increased hepcidin can result in inhibition of ferroportin preventing efflux of iron from the cell (9). Therapeutically, ferroptosis may be prevented using iron chelators, such as deferoxamine (DFO), which reduce the labile iron pool, or antioxidants, such as ferrostatin-1, which scavenge lipid radicals and limit ROS-induced membrane damage (5, 7).

### Osteoporosis

Osteoporosis is characterized by an imbalance between bone resorption and bone formation, leading to a progressive loss of both trabecular and cortical bone mass and density. This imbalance may result from increased osteoclastic resorption or decreased osteoblastic bone formation; however, in most cases, osteoporosis is predominantly driven by excessive osteoclast activity (16).Because the resorption phase of bone remodeling is shorter than the formation phase, such that even modest increases in resorption can lead to bone loss (17, 18), indicating that any increase in bone resorption will likely lead to loss of bone mass (18). A decrease in bone density results in a decrease in the weight-bearing ability of the bone, causing an increased risk of fractures, such as those in the vertebral column particularly in the high-risk population. The high-risk groups for osteoporosis are postmenopausal women (>50 years on average) and the elderly, especially those over 80 years old (16). In addition to estrogen deficiency in women, declining testosterone levels in aging men also contribute to osteoporosis risk (19). Generally, there are several risk factors for osteoporosis, with one of the most notable risks being decreased vitamin D (18). The active form of vitamin D, namely, 1,25-dihydroxycholecalciferol is important for increasing intestinal absorption and reducing renal tubular excretion of calcium and phosphate, as well as inhibiting the effects of parathyroid hormone, which induces osteoclastic activity (18). Lifestyle factors, such as physical inactivity and poor dietary consumption of calcium and protein, affect bone mineralization ability. Other factors, such as genetics, chronic alcoholism, smoking and, long-term use of medications (e.g., glucocorticoids, heparin, and L-thyroxine) can also contribute to osteoporosis development (16).

Osteoclast differentiation is primarily driven by the receptor activator of nuclear factor-κB (RANK) ligand (RANKL). RANKL binds to its receptor, namely, RANK, on osteoclast precursors and activates downstream signaling cascades, including nuclear factor kappa light chain enhancer of activated B cells (NF-κB) and nuclear factor of activated T cells 1 (NFATc1), which promote precursor differentiation into mature, bone-resorbing osteoclasts and enhance their resorptive activity. Inflammatory mediators, such as interleukin-6 (IL-6) and tumor necrosis factor-α (TNF-α), promote osteoclastogenesis; however, their actions are largely modulatory and commonly operate through the RANK–RANKL axis. For example, these mediators, increase RANKL expression in stromal/osteoblastic cells or by sensitizing precursors to RANKL stimulation (17, 18).

Estrogen plays a critical regulatory role in osteoclastogenesis primarily through modulation of the RANK–RANKL signaling pathway. RANKL, which is produced by osteoblasts, osteocytes, bone marrow stromal cells, and activated T and B lymphocytes, binds to RANK on osteoclast precursors to promote their differentiation into mature osteoclasts and to enhance osteoclastic activity via NF-κB signaling. Estrogen suppresses this process indirectly by stimulating osteoblasts to produce osteoprotegerin (OPG), a soluble decoy receptor that binds RANKL and prevents its interaction with RANK. In addition to regulating osteoclast differentiation and activity, estrogen directly controls osteoclast survival by promoting osteoclast apoptosis. Estrogen binding to estrogen receptor-α (ER-α) in osteoblasts induces the expression of Fas ligand (FasL), which activates the Fas/FasL extrinsic apoptotic pathway in osteoclasts, thereby limiting osteoclast lifespan. Estrogen has also been reported to enhance transforming growth factor-β (TGF-β) signaling, which further contributes to osteoclast apoptosis and coupling between bone resorption and formation (18). Consequently, estrogen deficiency leads to increased osteoclast differentiation, enhanced resorptive activity, and reduced osteoclast apoptosis, resulting in an imbalance between bone resorption and formation, ultimately driving postmenopausal bone loss (17, 19).

Studies related to runt-related transcription factor 1 (Runx1) and runt-related transcription factor 2 (Runx2) have increased the broadened our understanding of osteoblastic differentiation (20, 21). One study has reported that Runx1 deletion in osteoblast precursors and differentiating chondrocytes leads to the downregulation of various factors and signaling molecules, including Runx2, resulting in decreased bone density of long bones and skulls in Runx1 knockout mice (20). Other studies have reported that knockout of Runx2 leads to decreased osteoblastic differentiation. Unexpectedly, overexpression of Runx2 leads to the same outcome (21). However, further studies related to Runx1/2 and the roles that gene polymorphisms and mutations in the genes play in relation to osteoporosis are still needed.

With advancing age, multiple tissues, including bone, undergo progressive structural and functional changes that contribute to organ dysfunction, and bone is no exception. Aging bone marrow is characterized by increased marrow adiposity and impaired function of bone marrow-derived mesenchymal stromal cells (BMSCs), from which osteoblasts are derived. Dysfunction of BMSCs reduces their osteogenic differentiation and proliferative capacity, thereby compromising osteoblast formation and contributing to age-related declines in bone formation (19). Recent studies have suggested that the main offenders in osteoporosis are ROS. The decrease in estrogen and testosterone leaves osteoblast, osteoclasts and osteocytes susceptible to ROS damage (18, 19). When combined with age-associated alterations in the bone microenvironment, these changes disrupt the balance of bone remodeling and ultimately promote osteoporosis (19). Ferroptosis is one such mechanism that involves accumulation of ROS that leads to peroxidation and eventual cell death. Given its close relationship with oxidative stress and lipid peroxidation, ferroptosis may contribute more substantially to the pathogenesis of osteoporosis than previously appreciated.

## Method

**Figure 2:**
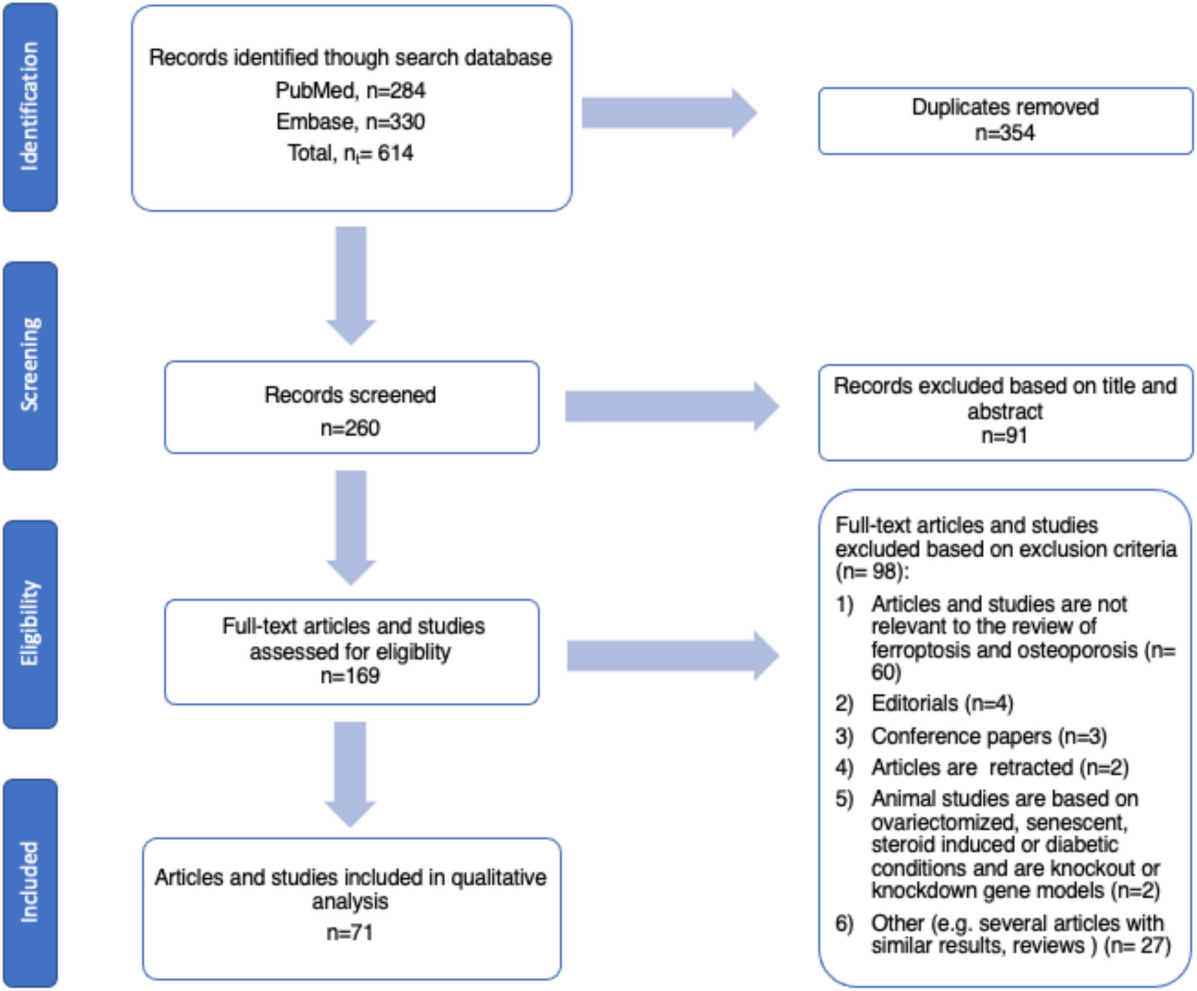
Flow chart of the systematic search process based on PRISMA guidelines.

As shown in **Figure 2**, a systematic review of recent research for ferroptosis and osteoporosis was conducted using the PubMed and Embase search engines from 1 January 1, 2019 to February 20, 2025. The following search terms were used: “ ferroptosis AND osteoporosis” or “ ferroptosis AND osteoblasts” or “ ferroptosis AND osteoclasts” or “ ferroptosis AND osteocytes”. The review was conducted following the PRISMA systematic search process (22). The relevant articles and studies were collected by two authors and reviewed by both. Exclusion and inclusion criteria were determined by both authors, and disagreements were resolved cooperatively. The eligibility criteria were as follows: 1) articles and studies relevant to topic of ferroptosis and osteoporosis; 2) studies published in 2019 onwards; 3) experimental studies with clear data and method outlines (e.g., cell lines and ferroptosis markers); and 4) animal studies based on ovariectomized, senescent, steroid induced, diabetic conditions, as well as are knockout or knockdown gene models.

## Results

### Role of ferroptosis in osteoblasts, osteoclasts, and osteocytes

Osteolineage cells refer to BMSCs, osteoblasts, and osteocytes, which represent sequential stages of osteogenic differentiation and play essential roles in bone formation and remodeling.

### BMSCs and osteoblasts

**Figure 3:**
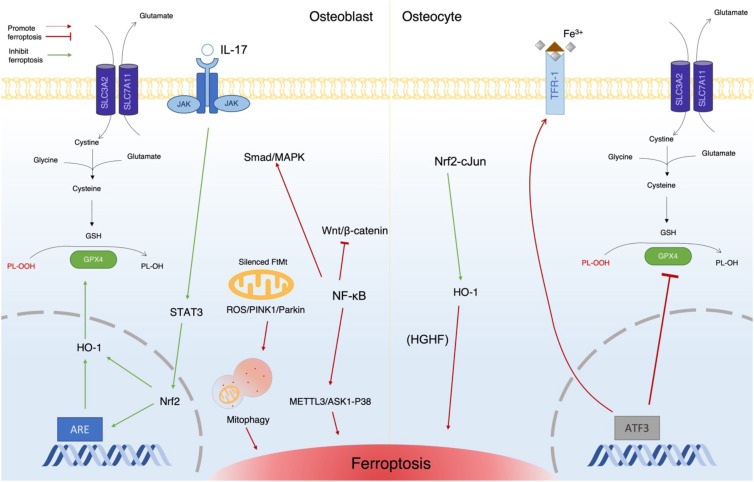
Diagram of the cellular events that promote or inhibit ferroptosis in osteoblasts and osteocytes. On the right, the diagram illustrates how ATF3 promotes ferroptosis in osteocytes by inhibiting GPX4 and upregulating TFR-1, as well as how HO-1 contributes to ferroptosis under HGHF conditions. In contrast, the Nrf2–cJun pathway inhibits ferroptosis in these cells. On the left, the diagram shows how IL-17 prevents ferroptosis in osteoblasts via ARE mediated upregulation of GPX4, alongside various other pathways that promote ferroptosis in osteoblasts. HGHF, high glucose high fat; HO-1, heme oxygenase 1; Nrf2, nuclear factor erythroid 2-related factor 2; ATF3, activating transcription factor 3; JAK, Janus kinase; STAT3, signal transducer and activator of transcription 3; NF-κB, nuclear factor kappa light chain enhancer of activated B cells; ARE, antioxidant-responsive elements.

Elevated iron levels have been reported to impair osteogenic differentiation of BMSCs and to induce ferroptosis in osteoblasts (**Figure 3**). Jiang et al. demonstrated that iron overload triggers ferroptosis in MC3T3-E1 cells, a murine osteoblast cell line, and that this process is attenuated by ferroptosis inhibitors (23). Mechanistically, iron overload suppresses osteoblastic differentiation by inhibiting the Wnt/β-catenin signaling pathway, which is critical for osteogenesis, while concurrently activating Smad and mitogen-activated protein kinase (MAPK) signaling pathways that negatively regulate osteoblast differentiation (24, 25). These signaling alterations result in reduced osteoblast maturation and decreased osteoblast numbers. In addition, iron overload–induced osteoblastic dysfunction is associated with downregulation of Runx2 in osteoprogenitor cells (26). Reduced Runx2 expression leads to impaired osteoblast differentiation and decreased expression of downstream osteogenic markers, including alkaline phosphatase and osteocalcin, ultimately resulting in diminished bone formation and bone mass loss (26). Nrf2 plays a critical role in maintaining redox homeostasis in mature osteoblasts, in part through activation of the Nrf2/HO-1 signaling pathway (13). In metabolic conditions such as type 2 diabetes mellitus (T2DM), suppression of Nrf2 signaling has been linked to increased susceptibility to ferroptosis (13). Bao et al. reported that interleukin-17 (IL-17) can modulates Nrf2 expression via activation of the Janus kinase 2 (JAK2)/signal transducer and activator of transcription 3 (STAT3) pathway, thereby alleviating ferroptosis in osteoblasts (27). Furthermore, recent evidence indicates that exposure to tobacco toxins induces ferroptosis in BMSCs through ferritinophagy (autophagic degradation of ferritin), highlighting that both exogenous and endogenous iron overload can promotes ferroptotic death of osteogenic cells (28).

### Osteocytes

Osteocytes are terminally differentiated osteoblasts embedded within the bone matrix, and they are the most abundant cell type in mineralized bone, playing a central role in maintaining skeletal homeostasis (29). Emerging evidence indicates that ferroptosis in osteocytes contributes to pathological bone loss. Jiang et al. demonstrated that genetic deletion of Nrf2 induces ferroptosis in osteocytes, which in turn enhances osteoclast activity; in addition, restoration of GPX4 expression is sufficient to prevent osteocyte ferroptosis in this model (30). Activating transcription factor 3 (ATF3) is also a key regulator of osteocyte ferroptosis. Yin et al. and Hong et al. reported that upregulation of ATF3 promotes iron uptake by TFR1 expression while simultaneously suppressing SLC7A11, thereby accelerating ferroptosis in senescent osteocytes and contributing to cortical bone loss (31, 32). These findings suggest a potential role for osteocyte ferroptosis in age-related osteoporosis.

The role of HO-1 in osteocyte ferroptosis is context dependent. Although its precise function remains incompletely defined, evidence from diabetic osteoporosis models suggests that HO-1 upregulation may protect against ferroptosis but that its inhibition exacerbates ferroptotic cell death (9, 14). Osteocyte ferroptosis has been reported to influence osteoclast activity. Jiang et al. reported that ferroptotic osteocytes exhibit increased expression of RANKL, leading to excessive osteoclast activation and bone loss in ovariectomized (OVX) mice (30). Similarly, Tang et al. demonstrated that osteocytes undergoing ferroptosis upregulate RANKL and IL-6, thereby enhancing osteoclast-mediated alveolar bone resorption in periodontitis (33). Together, these findings highlight osteocytes as a critical link between ferroptosis and osteoclast-driven bone resorption (**Figure 3**).

### Osteoclasts

**Figure 4:**
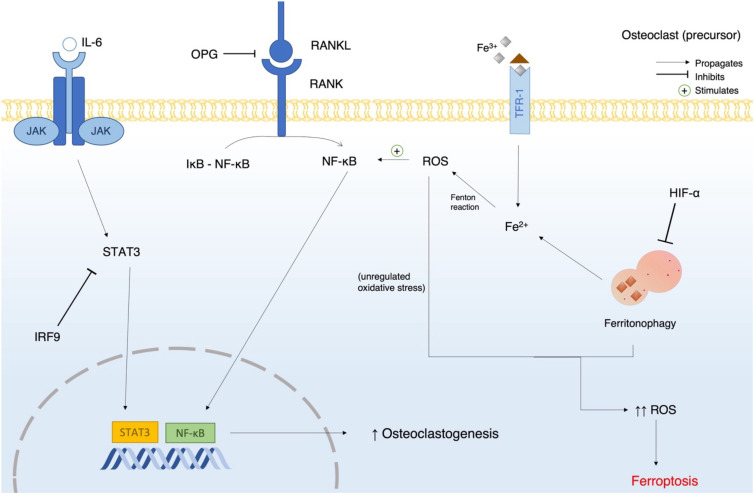
Diagram of the cellular events that promote osteoclastogenesis and ferroptosis during osteoclastic differentiation. The diagram illustrates how RANKL initiates osteoclastogenesis in a pre-osteoclast cell and how ROS produced via the Fenton reaction promotes this differentiation. When ROS production becomes unregulated, ferroptosis occurs. OPG, osteoprotegerin; IRF9, interferon regulatory factor 9; Janus kinase; STAT3, signal transducer and activator of transcription 3; NF-κB, nuclear factor kappa light chain enhancer of activated B cells; IκB, inhibitor of kappa B; HIF-α, hypoxia inducible factor alpha; ROS, reactive oxygen species.

Wang et al. reported that iron stimulation induces osteoclastic differentiation and bone resorption by activating the NF-κB signaling pathway through the generation of ROS, suggesting that ROS is a key mediator of osteoclast activity (34). Similarly, Ni et al. observed that under normoxic conditions, cells undergoing RANKL-induced osteoclast differentiation exhibited an iron-starvation response, as characterized by increased TFR1 expression and activation of ferritinophagy (35); they attributed this activation to downregulation of aconitase. Ni et al. also reported that the iron starvation response and ferritinophagy are not observed in hypoxic conditions. In addition, Ni et al. reported that within 2 days of the experiment, a modest number of cells subjected to normoxia exhibit decreased viability and undergo ferroptosis (cell death). Ni et al. hypothesized that the fate of monocytes during osteoclastogenesis may be decided by oxygen concentration and cellular density. Furthermore, in conditions where hypoxia-inducible factor-1 alpha (HIF-1a), which is a ferritinophagy inhibiting factor, is inhibited, Ni et al. observed a decreased rate of bone loss in OVX mice (35). ROS produced via ferritinophagy or iron-dependent ROS production have a role in RANKL-induced osteoclast differentiation, but unregulated ROS production can result in ferroptosis-mediated death. Moreover, ferritinophagy is mediated by nuclear receptor coactivator 4 (NCOA4), which can be inhibited by HIF-1a and endothelial cell (EC)-derived extracellular vesicles (Exos) (9). Qu et al. reported that myeloid zinc finger 1 (MZF1) is involved in RANKL-induced ferroptosis activity in osteoclasts; in MZF1 knockout mice, they reported that the expression of key osteoclast differentiation genes significantly increases and that ROS and intracellular iron decrease. Qu et al. concluded that MZF1 knockout increases Nrf2/GPX4 signaling pathway which improves cell viability (36). Regarding a role of mitochondrial transfer in osteoclastic ferroptosis, Ding et al. reported that osteolineage cells transfer mitochondria to myeloid cells, with monocytes/macrophages receiving the most mitochondria. This transfer is mediated by mitochondrial rho GTPase 1(MIRO1), and a deficiency in MIRO1 results in bone loss. Ding et al. hypothesized that transferred mitochondria influence GSH metabolism, which promotes ferroptosis-mediated death in osteoclasts, ultimately decreasing bone loss (37). Despite these findings (**Figure 4**), the precise role of ferroptosis in RANKL-induced osteoclast differentiation remains unclear and warrants further investigation.

### Age-related osteoporosis

Xu et al. demonstrated that the VDR activator 1,25(OH)_2_D_3_ prevents both ferroptosis and the D-gal–induced osteoblastic senescence program. Notably, treatment with either 1,25(OH)_2_D_3_ or the ferroptosis inhibitor ferrostatin-1 reduced the expression of senescence markers in D-gal–induced osteoblasts, suggesting that VDR activation suppresses osteoblastic senescence by inhibiting ferroptosis (38). Hu et al. summarized clinical and experimental evidence showing that circulating irisin increases with exercise, promotes osteoblastic differentiation, and is associated with lower fracture risk (39). In ischemia-reperfusion models, Wang et al. Demonstrated that irisin activates the Nrf2/HO-1 signaling axis, suppresses ferroptosis, and preserves mitochondrial function, supporting an anti-ferroptotic protective effect relevant to aging bone (40). Bone marrow adipocyte accumulation has also been implicated in age-related osteoporosis. Huang et al. reported that osteoblasts in adipocyte-conditioned media exhibit increased levels of intracellular lipid droplet accumulation, increased expression of ferroptosis-related genes, decreased oxidative phosphorylation, and decreased differentiation (41). Exposure to various environmental stressors, such as tobacco toxins, induces ferritinophagy and ferroptosis in BMSCs, which increases labile iron and ROS, thereby impairing osteoblastic differentiation—providing a mechanistic basis for smoking-associated age-related bone loss (28). Collectively, these studies indicate that vitamin D signaling (VDR/Nrf2/GPX4), irisin (Nrf2/HO-1), marrow adiposity, and tobacco exposure converge on iron dysregulation, lipid peroxidation, and ferroptosis in osteogenic cells; thus, these components should be considered when evaluating interventions to mitigate age-related osteoporosis.

### Postmenopausal osteoporosis

Osteoporosis factors related to aging are also implicated in postmenopausal osteoporosis (16). Epigenetic modifications play a prominent role in age-related bone loss and are particularly relevant in postmenopausal osteoporosis (42, 43). DNA methylation is involved in decreased bone density. For example, Jintaridth et al. reported that *Alu* hypomethylation in blood cells is associated with decreased bone density in postmenopausal women (43). In OVX mice, Ruan et al. demonstrated that increased expression of DNA methyltransferases (DNMTs) leads to hypermethylation of the GPX4 promoter in osteoblasts, contributing to ferroptosis and osteoporosis (44). Although these findings were observed in osteoblasts when iron was administered, GPX4 suppression was not observed in osteoclasts. When DNMT inhibitor was administered, hypermethylation of the promoter was reversed and GPX4 was expressed; Ruan et al. proposed that DNMT inhibition may be a new target for osteoporosis treatment (44). In OVX mice, Lan et al. reported that hyperactive osteoclasts have decreased expression of interferon regulatory factor 9 (IRF9) (45). To discover the role of IRF9 in osteoclasts, Lan et al. knocked down IRF9 in OVX mice, which decreased lipid peroxidation, increased mitochondrial activity and upregulated GPX4, and upregulated ferritin heavy chain 1 (FTH1), a ferritin storage protein; they concluded that decreased IRF9 expression activates the STAT3, which upregulates certain factors, such as GPX4 (45). Although findings from OVX rodent models are useful to model estrogen-deficiency–induced bone loss, they have inherent limitations and cannot be directly extrapolated to postmenopausal women; therefore, translational and clinical studies are needed to confirm preclinical observations in human populations (46, 47). Given that interferon regulatory factors play a central role in adaptive and innate immune responses, these findings indicate that immune dysregulation and chronic inflammation may contribute to postmenopausal osteoporosis by modulating ferroptosis in osteoclasts (19, 48).

### Diabetic osteoporosis

T2DM is characterized by insulin resistance and hyperglycemia. Irregularities in insulin and glucose metabolism cause abnormalities, such as hyperlipidemia, which result in diabetic complications, including cardiovascular disease and osteoporosis. A diabetic environment consisting of high glucose and high fat (HGHF) leads to osteogenic cell dysfunction by increasing ferritin expression, increasing TFR1 expression, and decreasing ferroportin expression (4, 49).

Recent studies have identified several factors that promote ferroptosis in osteoblasts under diabetic conditions. In high-fat diet (HFD)-/streptozotocin (STZ)-induced type 2 diabetic C57BL/6J mice (male), Wei et al. reported that asperosaponin VI (AVI) restores GPX4 expression by inhibiting DNMT1/3a-mediated GPX4 promoter hypermethylation, thereby reducing osteoblast ferroptosis and ameliorating diabetic osteoporosis (50). Peng et al. reported that elevated tissue inhibitor of metalloproteinase 1 (TIMP1) in T2DM promotes ferroptosis by inhibiting TFR1 ubiquitination (51). Du et al. reported that acid sphingomyelinase promotes ferroptosis by activating autophagic degradation of GPX4 (52), and Ren et al. reported that ELAV1 induces ferroptosis by increasing expression of divalent metal transporter 1 (DMT1) (53). High glucose and fat also enhance ferroptosis in osteocytes, which can be attenuated through activation of the Nrf2/HO-1 pathway (54). This pathway can be pharmacologically upregulated by agents, such as melatonin or maresin1, which alleviate high glucose-induced ferroptosis both *in vitro* and *in vivo* (55). Furthermore, vitamin K2 prevents ferroptosis in BMSCs in T2DM by activating the AMP-activated protein kinase (AMPK)/sirtuin 1 (SIRT1) pathway, which enhances GPX4 activity (56).

### Steroid-induced osteoporosis

The mechanism underlying steroid-induced osteoporosis (SIOP) remains incompletely understood. A hallmark of SIOP is a reduction in osteoblast numbers (57, 58). High doses of dexamethasone have been reported to induce ferroptosis in osteoblasts (7, 59). Sun et al. reported that steroids promote ferroptosis through increased expression of p53, which downregulates GPX4 and system x_c_^−^ (60). Li et al. reported similar findings but also observed downregulation of ferroptosis suppressor protein 1(FSP1) and upregulation of ACSL4 (57). In addition, Ding et al. demonstrated that glucocorticoid therapy decreases mitochondrial transfer between osteolineage and myeloid lineage cells, which is associated with increased bone loss (37), suggesting that impaired mitochondrial communication may contribute to SIOP. Emerging evidence indicates that EC-Exos can ameliorate SIOP. EC-Exos contain functional proteins, bioactive lipids, mRNAs, long non-coding RNAs, and microRNAs (61, 62). micro-RNAs have anti-ferroptotic effects in melanoma (61), and the microRNA-mediated anti-ferroptotic effects occur through Nrf2 regulation in steroid-induced femoral head osteonecrosis (63). In addition, Yang et al. reported that EC-Exo can inhibit ferritinophagy in osteoblasts (62). However, further studies are needed to elucidate the precise mechanisms underlying EC-Exos mediated protection in SIOP.

### Obesity-related osteoporosis

Obesity causes adipocyte accumulation in bone marrow, which leads to osteoprogenitor cell dysfunction in aging (41, 64). Mechanistically, a HFD has been reported to suppress the expression of SLC7A11 and GPX4, and this downregulation correlates with elevated serum TNF-α levels. Chen et al. reported that obese rats exhibited increased TNF-α levels alongside reduced bone volume, trabecular number, and cortical thickness. Moreover, administration of ferroptosis inhibitors decreases TNF-α levels and ameliorates bone loss (64), suggesting that ferroptosis may mediate obesity-induced skeletal deterioration.

### Hereditary hemochromatosis and osteoporosis

Beyond metabolic disorders such as diabetes mellitus and obesity, genetically determined diseases characterized by systemic iron overload may provide further insight into the role of ferroptosis in bone pathology. Hereditary hemochromatosis, which is caused by mutations in the HFE gene, leads to excessive intestinal iron absorption and progressive iron deposition in multiple organs, including bone. Clinical and experimental studies have reported reduced bone mineral density and increased fracture risk in patients with iron overload, suggesting a direct detrimental effect of excess iron on skeletal homeostasis. From a mechanistic perspective, chronic iron accumulation creates a permissive environment for ferroptosis by increasing the labile iron pool, promoting lipid peroxidation, and overwhelming antioxidant defense systems such as the GPX4–glutathione axis. Osteoblasts and osteocytes, which are highly sensitive to oxidative stress, may be particularly vulnerable to iron-induced ferroptotic damage, leading to impaired bone formation and compromised bone quality. Although direct evidence linking ferroptosis to bone loss in hereditary hemochromatosis remains limited, this condition represents a compelling human model supporting the pathogenic role of iron-driven ferroptosis in osteoporosis (65, 66).

### Targeting ferroptosis as a therapeutic strategy for osteoporosis

Accumulating evidence implicates ferroptosis in osteoporosis, and therapeutic strategies targeting this pathway have gained attention. **Table 1** summarizes pharmacological and experimental interventions affecting osteoblasts, osteocytes, and osteoclasts.

**Table 1:** Proposed treatment strategies and therapies.

Therapeutic product/method	Mechanism of action	Effect on cells	Reference
Iron chelators (e.g., DFO-1)	Binds to free iron	Prevents ferroptosis in osteoblasts and osteoclasts	(5, 7)
Antioxidants (e.g., Fer-1, quercetin, and vitamin E)	Inhibits lipid peroxidation by upregulating GSH, GPX4 and SLC7A11	Prevents accumulation of lipid peroxides in osteoblasts and osteoclasts	(9, 67)
Melatonin	Increases Runx2, inhibits PPARy, and inhibits RANKL expression in osteoblasts. Activates P13K/AKT/mTOR signaling pathway in BMSCs	Promotes osteoblast differentiation. Attenuates SIOP	(9, 57)
Vitamin D analogs (e.g., eldecalcitol)	Vitamin D receptor stimulates the Nrf2/GPX4 signaling pathway	Prevents of ferroptosis in senescent osteoblasts and osteocytes	(38, 86)
Vitamin K2	Activates the AMPK/SIRT1 signaling pathway promoting GPX4 activity	Prevents ferroptosis of BMSCs in T2DM	(56)
EC-Exos	–	Reverses steroid induced osteoporosis	(61, 62)
Irisin	–	Decreases fracture risk and prevents osteoblast ferroptosis	(39, 83)
Aconine	Upregulates GPX4 and suppresses ACSL4 through inhibition of phosphorylation of I-kB and p65 in the NF-kB signaling pathway	Attenuates osteoclast-mediated bone resorption and regulates ferroptosis	(89)
Resveratrol	Upregulates SLC7A11 and GPX4	Prevents osteocyte ferroptosis in diabetic periodontitis	(82)
Icariin	Activates the Nrf2/HO-1 signaling pathway	Prevents osteoblast ferroptosis	(68)
Mangiferin	Activates Keap1/Nrf2/SLC7A11/GPX4	Prevents osteoblast ferroptosis	(69)
Maresin 1	Upregulates the Nrf2/GPX4 signaling pathway and SLC7A11	Prevents osteoblast ferroptosis in T2DM	(55)
Biochanin A	Upregulates the Nrf2/system x_c_^-/^GPX4 signaling pathway	Inhibits osteoclasts differentiation and prevents bone loss	(80, 81)
Curcumin	Regulates the AKT/GSK3b signaling pathway to improve mitochondrial activity	Prevented osteoblast death by oxidative stress	(81)
Poliumoside	Activates the Nrf2/GPX4 signaling pathway	Prevents ferroptosis in BMSCs	(77)
Fraxin	Activates the Nrf2/GPX4 signaling pathway	Prevents osteoblast ferroptosis in SIOP	(70)
Fructus *Ligustri Lucidi*	Activates the Nrf2/HO-1 signaling pathway	Prevents ferroptosis and bone loss in PMOP	(71)
Qing’e pill	Regulates ATM and AKT/PI3K pathway	Prevents osteoblast ferroptosis	(72)
Picein	Activates the Nrf2/HO-1/GPX4 signaling pathway	Prevents ferroptosis in BMSCs	(74)
Polycytosine RNA-binding protein 1	Iron ion chaperone that binds iron and transfers to ferritin	Prevents osteoblast ferroptosis in T2DM	(84)
Aucubin	Activates the BMP2/Smads signaling pathway	Stimulates proliferation and differentiation of BMSCs	(78)
Artesunate	Inhibits iron uptake stimulated by osteoclast differentiation	Inhibits bone resorption	(90)
Sarsasapogenin	Activates the GPX4/SLIT3/ROBO1 signaling pathway	Prevents ferroptosis of BMSCs in PMOP	(79)
Astaxanthin	Activates the JAK2/SATA3 signaling pathway	Prevents osteoblast ferroptosis in SI osteonecrosis	(73)
Isovitexin	Upregulates SIRT3 which suppresses mitophagy	Prevents osteoblast ferroptosis in SI osteonecrosis	(75)
Arecoline	Activates HO-1	Inhibits ferroptosis and osteoblast differentiation suppression	(76)
Asperosaponin VI	Inhibits DNMT to alleviate GPX4 promoter suppression	Prevents osteoblast ferroptosis	(50)
YBX1 protein upregulation	Activates ATF4/FSP1 signaling pathway	Prevents osteoblast ferroptosis	(85)
Zoledronic acid	Inhibits FBXO9-mediated p53 ubiquitination	Stimulates osteoclast ferroptosis	(87)
Saikosaponin A	Inhibits osteoclastogenesis	Stimulates osteoclast ferroptosis	(88)
Zanthoxylum bungeanum Maxim	Inhibits osteoclastogenesis by suppressing the ERK/c-JUN/NFATc1 signaling pathway	Stimulates osteoclast ferroptosis	(91)

DFO-1, Deferoxamine 1; Fer-1, Ferroptosis inhibitor 1.

### Osteoblasts and BMSCs

Most therapeutic strategies targeting osteoblasts and BMSCs aim to limit labile iron accumulation, suppress lipid peroxidation, or enhance endogenous antioxidant defenses. Iron chelators such as deferoxamine (DFO), reduce the labile iron pool and prevent ferroptosis in osteoblasts and osteoclasts in preclinical models (5, 7). Small-molecule ferroptosis inhibitors and antioxidants, including ferrostatin-1, quercetin, and vitamin E, preserve osteoblast viability by inhibiting lipid peroxidation and enhancing the glutathione–GPX4/system xc^-^ axis under oxidative or diabetic conditions (9, 67).

Multiple bioactive compounds, including icariin, mangiferin, fraxin, poliumoside, picein, maresin-1, vitamin K2, and vitamin D analogs, promote osteogenic differentiation and suppress ferroptosis through activation of Nrf2/HO-1 signaling and restoration of Runx2-dependent programs (38, 39, 55–57, 61, 62, 68–74). Other agents, such as curcumin, biochanin A, aucubin, and sarsasapogenin, reduce ferroptotic vulnerability via antioxidant, epigenetic, or mitochondrial mechanisms in experimental osteoporosis models (50, 75–81).

Several regulators of iron metabolism and ferroptosis have also been identified in osteoblast lineage cells. Resveratrol alleviates diabetic osteoporosis by activating the SLC7A11/GPX4 axis (82), while FNDC5/irisin, polycytosine RNA-binding protein 1 (PCBP1), and Y-box binding protein 1 (YBX1) protect osteoblasts from ferroptosis and enhance osteogenic potential through modulation of ferroptosis-related pathways and intracellular iron homeostasis (83–85).

### Osteocytes

Given their longevity and sensitivity to oxidative stress, maintaining redox balance and iron homeostasis in osteocytes is critical for bone integrity. Upregulation of GPX4, activation of Nrf2 signaling, and increased HO-1 activity protect osteocytes from ferroptosis in diabetic and aging models, and certain agents, such as eldecalcitol and resveratrol, exhibit protective effects (38, 82, 86). In addition, limiting ferritinophagy and intracellular iron accumulation in osteocytes suppresses osteocyte-derived RANKL and IL-6 signaling, which indirectly represses osteoclastogenesis (9, 14).

### Osteoclasts

Therapeutic modulation of ferroptosis in osteoclasts focuses primarily on suppressing excessive bone resorption by selectively inducing ferroptotic cell death. Several compounds, including artesunate, aconine, and saikosaponin A, inhibit osteoclast differentiation and activity by disrupting iron metabolism and inducing ferroptosis (87–90). Conventional antiresorptive agents such as zoledronic acid, have also been reported to promote osteoclast ferroptosis in preclinical models. Additionally, modulators targeting NF-κB/ERK signaling or mitochondrial iron handling further reduce osteoclast-mediated bone loss (89, 91).

## Discussion

**Figure 5:**
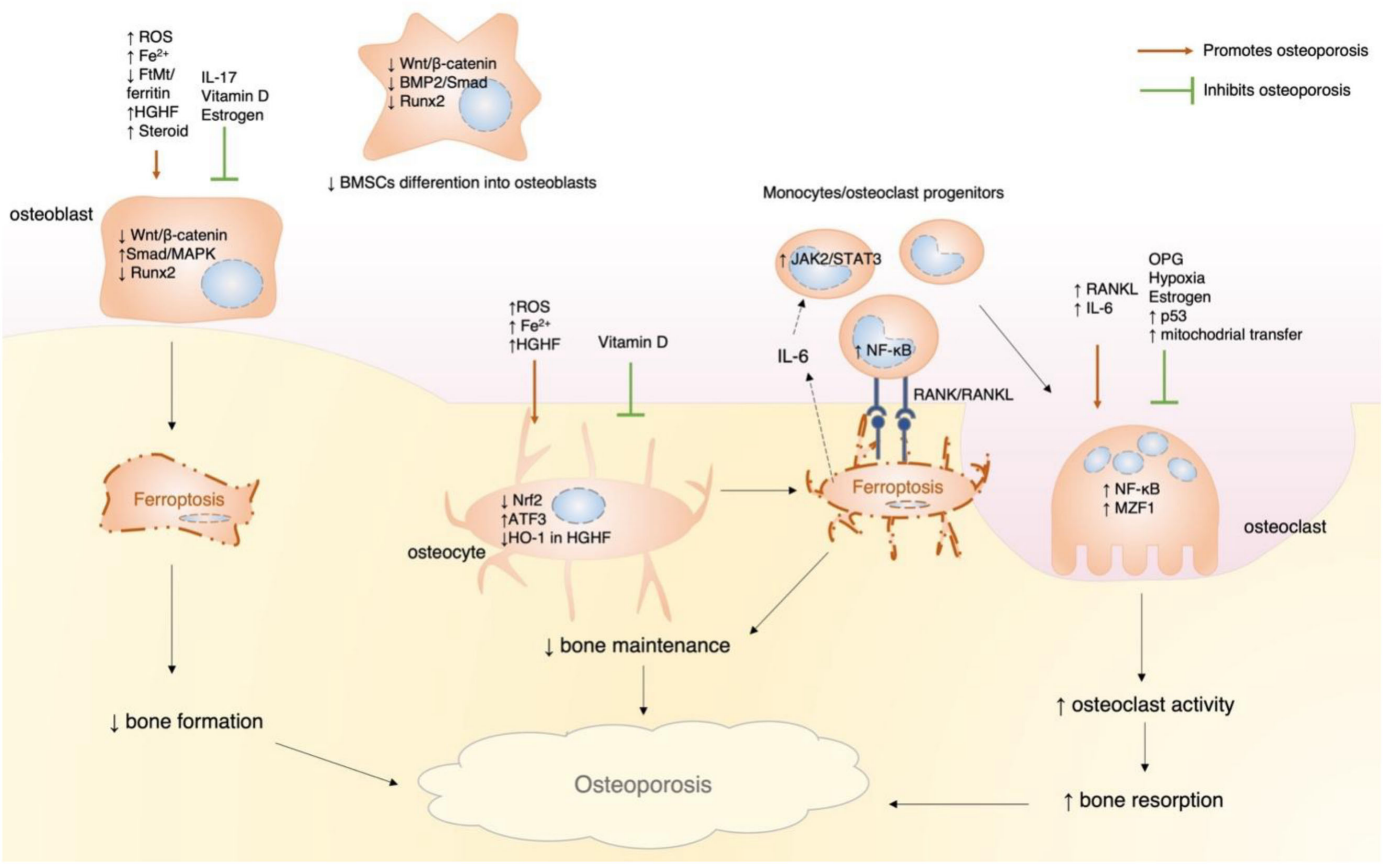
Diagram showing how osteoporosis develops through ferroptosis. The process begins with the ferroptotic death of osteoblasts, which decreases bone formation. This is followed by decreased bone maintenance due to osteocyte ferroptosis. Finally, increased osteoclastic activity is driven by RANKL and IL-6, which are released from ferroptotic osteocytes, leading to elevated bone resorption and net bone loss. ROS, reactive oxygen species; FtMt, mitochondrial ferritin; HGHF, high glucose high fat; OPG, osteoprotegerin; MZF1, myeloid zinc finger 1.

The prevention of osteoporosis requires a balance of bone resorption and bone formation. Disruption of this delicate balance due to various factors, such as iron overload, senescence, and hormones (estrogen and glucocorticoid), can lead to osteoporosis (18, 19, 26). Ferroptosis, as a key regulator of iron-mediated cell death, has emerged as an important factor in this process (**Figure 5**). Therefore, elucidating the mechanistic involvement of ferroptosis and current research limitations is essential for guiding the development of targeted therapeutic and preventive strategies.

### Mechanistic insights and knowledge gaps of ferroptosis in osteoporosis

Significant progress has been made in understanding how ferroptosis affects osteogenic cells; however, there are still various aspects that remain incompletely understood. One key area is the association between ferroptosis and RANKL-induced differentiation in osteoclast progenitors. The initiation of an iron starvation response and ferritinophagy during RANKL stimulation under normoxic conditions suggests that iron and ROS play a critical role in osteoclast differentiation (35). Understanding this association may provide the full scope of the osteoclast differentiation pathway and insight into target pathways for future treatment strategies. Lan et al. reported that decreased IRF9 expression increases osteoclast activity through JAK2/STAT3 signaling pathway activation (45). The JAK2/STAT3 pathway is activated by cytokines particularly IL-6 (92). Moreover, osteocytes secrete IL-6 and RANKL during ferroptosis (33), suggesting that they may play a larger role in osteoporotic processes than previously recognized. To date, studies examining how ferroptosis mediates osteocyte death and its impact on bone homeostasis remain scarce. Hormonal and inflammatory dysregulation are also implicated in osteoporosis, particularly in women. Lifelong hormonal fluctuations—from menarche to menopause—alongside increased susceptibility to chronic inflammatory diseases, contribute to elevated osteoporosis risk post menopause (17, 93). In chronic inflammatory diseases, acute phase reactants, such as hepcidin, are chronically elevated; hepcidin production is stimulated by the IL-6 cytokine (94–96). Because hepcidin influences ferroptosis activity (9), its effect on osteoporosis in women with inflammatory disease should be further studied. Menstruation and pregnancy affect iron levels, and several factors, such as menorrhagia and amenorrhea, resulting in depleted or accumulated iron reserves (97). The implications of ferroptosis in women with irregular menstrual history or multipara and how ferroptosis can influence onset of osteoporosis in the postmenopausal period should be investigated. Finally, genetic disorders that impair antioxidant defenses, such as glucose-6-phosphate dehydrogenase (G6PD) deficiency, may modulate susceptibility to ferroptosis in osteogenic cells. Further studies in this area are needed to elucidate how these conditions influence osteoporosis development (98).

## Conclusion

In summary, research on ferroptosis in osteoporosis is still in its early stages, with most studies conducted in animal models. Further investigation into ferroptosis-mediated osteoporosis in humans, particularly using human cell lines, is urgently needed. Current findings have provided valuable insights into the mechanisms and potential targets in osteogenic cells. These advances offer a promising foundation for the development of novel therapeutic strategies for osteoporosis.

## Data Availability

The original contributions presented in the study are included in the article/supplementary material. Further inquiries can be directed to the corresponding author.
